# Angiotensin II Induces Vascular Endothelial Dysfunction by Promoting Lipid Peroxidation-Mediated Ferroptosis via CD36

**DOI:** 10.3390/biom14111456

**Published:** 2024-11-17

**Authors:** Qian Zhou, Ying Zhang, Wei Shi, Lu Lu, Jianglan Wei, Jinhan Wang, Hu Zhang, Yuepu Pu, Lihong Yin

**Affiliations:** 1 Key Laboratory of Environmental Medicine Engineering, Ministry of Education, School of Public Health, Southeast University, Nanjing 210009, China; 230229470@seu.edu.cn (Q.Z.); 101300315@seu.edu.cn (Y.Z.); 230239085@seu.edu.cn (W.S.); 230229018@seu.edu.cn (L.L.); 220223650@seu.edu.cn (J.W.); 220223622@seu.edu.cn (J.W.); yppu@seu.edu.cn (Y.P.); 2 School of Public Health, Yangzhou University, Yangzhou 225009, China; zhanghu@yzu.edu.cn

**Keywords:** Ang Ⅱ, HUVECs, CD36, endothelial dysfunction, ferroptosis

## Abstract

Angiotensin II (Ang II) is an effective vasoconstriction peptide, a major effector molecule of the renin–angiotensin–aldosterone system (RAAS) and one of the important causes of endothelial dysfunction. Ferroptosis is considered to be involved in the occurrence and development of cardiovascular diseases. This study is dedicated to exploring the role and mechanism of Ang II-induced ferroptosis in HUVECs and to finding molecular targets for vascular endothelial injury and dysfunction during the progression of hypertension. In this study, we found that with the increase in exposure concentration, the intracellular ROS content and apoptosis rate increased significantly, the NO release decreased significantly in the medium- and high-concentration groups and the ET-1 content in the high-concentration group increased significantly. The expression of ZO-1 protein was significantly decreased in the high-concentration group. The expression of p-eNOS, VE-cadherin and Occludin protein showed a dose-dependent downward trend, while the ICAM-1 protein showed an upward trend. Ang II caused lipid metabolism disorders in HUVECs, and the PL–PUFAs associated with ferroptosis were significantly increased. In addition, Ang II promoted a significant increase in intracellular free Fe^2+^ content and MDA and a significant decrease in GSH content. Furthermore, the expression of GPX4, SLC7A11 and SLC3A2 was down-regulated, the expression of ACSL4, LPCAT3 and ALOX15 was up-regulated, and the ratio of p-cPLA2/cPLA2 was increased. After the intervention of ferroptosis inhibitor Fer-1, the injury and dysfunction of HUVECs induced by Ang II were significantly rescued. Immunofluorescence results showed that the expression of CD36 showed a significant increasing trend and was localized in the cytoplasm. Over-expression of CD36 promoted Ang II-induced ferroptosis and endothelial dysfunction. In conclusion, Ang II induces the injury of HUVECs, decreases vascular diastole and endothelial barrier-related molecules, and increases vascular constriction and adhesion-related molecules, which may be related to CD36 and its mediated lipid peroxidation and ferroptosis signals.

## 1. Introduction

The renin–angiotensin–aldosterone system (RAAS) is an important endocrine system in the human body that is mainly responsible for the regulation of blood pressure [1]. Angiotensin II (Ang II), as a crucial effector molecule of the RAAS system, can cause vasoconstriction and increase peripheral circulation resistance, leading to an increase in blood pressure. Hypertension is an important metabolic risk factor for various cardiovascular diseases (CVDs). Endothelial dysfunction (ED) is closely related to the pathophysiology of hypertension and the occurrence or development of CVD induced by hypertension [2]. Accumulating evidence suggests that Ang II is a pro-inflammatory mediator of hypertension by generating ROS through endothelial cells (ECs) [3,4]. At present, there are also a large number of studies using Ang II-exposed human umbilical vein endothelial cells (HUVECs) to construct a model of hypertensive vascular endothelial dysfunction (VED) [5,6].

Ferroptosis is a new type of cell death mechanism, mainly mediated by iron-dependent lipid peroxidation [7,8,9], which is closely related to the metabolic disorder of polyunsaturated fatty acids (PUFAs) [10]. Glutathione peroxidase 4 (GPX4) is an important monitoring mechanism for inhibiting ferroptosis [11]; the degradation of GPX4 can lead to the accumulation of lipid reactive oxygen species (ROS) [12]. In addition, free PUFAs can be esterified into PL–PUFAs catalyzed by Acyl-CoA synthetase long-chain ligase (ACSL) and lysophosphatidyl-CoA acyltransferases (LPCAT) and further oxidized by arachidonate lipoxygenase (ALOX) to accumulate PLOOH [10]. The accumulation of lipid peroxidation and the inactivation of the antioxidant system eventually lead to cell death. There is evidence that ferroptosis mediates the pathogenesis and progression of a variety of CVDs [13].

CD36, also known as fatty acid translocase (FAT), belongs to the B2 scavenger receptor family [14]. CD36 has been reported to be a pattern recognition receptor widely expressed on various types of cells, including endothelial cells, adipocytes, cardiomyocytes, hepatocytes, renal tubular epithelial cells, monocytes, and macrophages [15,16,17], and to be involved in apoptotic cell uptake, signal transduction, cell adhesion, angiogenesis, and immune function. More importantly, it plays an important role in lipid metabolism and promotes the transport of long-chain fatty acids (FAs), phospholipids, and oxidized low-density lipoproteins (LDLs) [18]. Ma et al. found that CD36 regulates ferroptosis in CD8^+^ T cells [19]. A recent study proved that 1-palmitoyl-2-glutaroyl-sn-glycero-3-phosphocholine (PGPC) induces ferroptosis of vascular endothelial cells through CD36, thereby impairing endothelial function [20].

In recent years, a number of studies have revealed the close relationship between ferroptosis and the pathogenesis of hypertension [21,22] and provided in vivo evidence that ferroptosis leads to endothelial dysfunction and atherosclerosis [23]. However, there is currently a lack of direct in vitro evidence and molecular mechanisms of hypertension-induced endothelial ferroptosis. This study aims to provide new insights into the molecular mechanism of hypertension-induced endothelial dysfunction, reveal the role and mechanism of ferroptosis in hypertension-induced vascular endothelial dysfunction, and find molecular targets for vascular endothelial injury and dysfunction during the progression of hypertension.

## 2. Materials and Methods

### 2.1. Reagents

Angiotensin II (Ang Ⅱ, C_50_H_71_N_13_O_12_, CAS No. 4474-91-3, purity ≥ 98%, molecular weight 1046.18, Cat. No. A9290) was purchased from Solarbio Technology Co., Ltd. (Beijing, China). The Ang II powder was dissolved into a 10 mM stock solution using autoclaved and filtered pure water, and the stock solution was stored at −80 °C after aliquoting. Ferrostatin-1 (Fer-1, C_15_H_22_N_2_O_2_, 10 mM, CAS No. 347174-05-4, purity > 99%, molecular weight 262.35, Cat. No. HY-100579) was purchased from MedChemExpress Biotechnology Co., Ltd. (Shanghai, China).

### 2.2. Cell Culture and Treatment

Human umbilical vein endothelial cells (HUVECs) were purchased from Zhongqiao Xinzhou Biotechnology Co., Ltd. (Shanghai, China). The HUVECs were cultured in a complete medium at 37 °C in a 5% CO_2_ incubator. The complete medium was a mixture of Dulbecco’s Modified Eagle Medium (DMEM), penicillin (100 µg/mL, Sigma, Burlington, MA, USA), streptomycin (100 µg/mL, Sigma, USA), and 10% fetal bovine serum (FBS) (Gibco, Courtaboeuf, France). Cells growing to passages 4 to 6 were used for further experiments. The HUVECs were plated in 6-well, 24-well, or 96-well plates and grown in DMEM containing penicillin and streptomycin with 1% FBS. When cells were close to 70–80% confluence, they were exposed to low, medium, and high concentrations (0.1, 10, and 100 μM) of Ang Ⅱ (dissolved by DMEM containing penicillin and streptomycin without FBS).

### 2.3. Cell Viability Assay

The Cell Counting Kit-8 (CCK-8, Beyotime Biotechnology, Shanghai, China) was used to determine the cytotoxicity after being exposed to 0.1, 1, 10 or 100 μM Ang Ⅱ for 3, 6, 12, 24, 36, 48 and 60 h, to observe the concentration and time-dependent effects. The CCK-8 detection solution was prepared according to the ratio of DMEM to CCK-8 solution 10:1, and 110 μL detection solution was added to a 96-well plate. After incubation at 37 °C for 2 h, the absorbance value of each well at 450 nm was recorded by a microplate reader (Biotek, Winooski, VT, USA). Cell viability (%) was calculated according to the following formula: [A(Well to be detected)-A(Blank)]/[A(Control)-A(Blank)]. A(Well to be detected): absorbance values of wells with treatment group cells and CCK-8 detection solution). A(Blank): absorbance values of wells with CCK-8 detection solution. A(Control): absorbance values of wells with control group cells and CCK-8 detection solution.

### 2.4. Measurement of Reactive Oxygen Species (ROS)

The intracellular Reactive Oxygen Species (ROS) levels were detected by a ROS Assay Kit (Beyotime Biotechnology, Shanghai, China). A ROS detection solution was prepared by the dichloro-dihydro-fluorescein diacetate (DCFH-DA) probe and DMEM (without FBS) in a ratio of 1:1000. After exposure to low, medium and high concentration Ang Ⅱ for 48 h; the HUVECs were incubated with detection solution for 30 min at 37 °C in a 5% CO_2_ incubator. Next, the cells were observed using fluorescence microscopy (Zeiss AX10, Oberkochen, Germany) and a flow cytometry system (Becton Dickinson Bioscience, Franklin Lakes, NJ, USA).

### 2.5. Cell Apoptosis Assessment

The cell apoptosis rate (%) was assessed by an Apoptosis Assay Kit (MeilunBio, Guangzhou, China). HUVECs were collected using trypsin without EDTA and then stained with Annexin V (FITC) and Propidium Iodide (PI) working solutions according to the manufacturer’s instruction manual. The cells were incubated at room temperature for 5 min away from light. The cell apoptosis rate (%) was measured by flow cytometry (Becton Dickinson Bioscience, USA), and the data were analyzed quantitatively and visually using FlowJo V10 software.

### 2.6. Nitric Oxide (NO) Release Detection

The cells in the logarithmic growth phase were plated on the six-well plate at a density of 1.5 × 10^4^/well. After Ang Ⅱ exposure for 48 h, the culture medium was collected, and the supernatant was taken as the sample to be measured after centrifugation. The nitric oxide content was determined by NO Assay Kit (Jiancheng Bioengineering Research Institute, Nanjing, China). According to the manufacturer’s operating manual, different detection reagents were added, and a sodium nitrite standard curve was prepared. After adding the color developer for 15 min, the OD values of each well were detected at 550 nm, and the NO content of the samples to be tested was calculated based on the standard curve.

### 2.7. Enzyme-Linked Immunosorbent Assay (ELISA) of ET-1

The cells were collected and suspended by PBS buffer solution, and then they were repeatedly frozen and thawed three times in a liquid nitrogen container and 37 °C water bath to fully release the cell contents. After centrifugation, the supernatant was taken as the sample to be tested. The ET-1 content in cells was determined using an ELISA kit (SenBeiJia Biological Technology, Nanjing, China) according to the manufacturer’s instructions.

### 2.8. Cellular Non-Targeted Metabolomics

Six parallels were set up in the low, medium and high concentration groups. After 48 h Ang Ⅱ treatment, the cells were washed with pre-cooled 0.9% sodium chloride and cooled on the ice containing 30% calcium chloride. Precooled methanol was added to the culture dishes, and the cells were collected with a scraper into Eppendorf tubes. After centrifugation, the supernatant was collected into the new Eppendorf tubes. The cell precipitate was added with 80% methanol. After grinding, it was repeatedly frozen and thawed in a liquid nitrogen tank three times. Then, after centrifugation, the supernatant obtained twice was collected into a 5 mL nitrogen blowpipe and slowly blown to dryness with nitrogen. The metabolites were redissolved in 80% methanol, centrifuged three times, and subjected to non-targeted metabolomics analysis on a UHPLC system equipped with an AB SCIEX Triple TOF 5600 Mass Spectrometer (Framingham, MA, USA). The raw data were converted by MSconvert (Version 3.0.23213-ff69fa9). R software (Version 4.1.2) was used to extract the peaks, retention time and metabolite mass-to-charge ratio of the original data. After standardization by EXCEL, the principal component analysis (PCA) model and orthogonal projection to latent structure discriminate analysis (OPLS-DA) model were established by SIMCA-P (Version 14.1). According to OPLS-DA analysis, metabolites with VIP score > 1 and significant differences between multiple groups (*p* < 0.05) were obtained. The Human Metabolome Database (HMDB, Version 5.0, https://hmdb.ca/, accessed on 1 October 2023) was further used to compare the secondary structure of the significantly differential metabolites (SDMs) to determine the name and type of the metabolites. Based on the SDMs, the heat map was drawn and the KEGG metabolic pathway was enriched by MetaboAnalyst (Version 6.0, https://new.metaboanalyst.ca/ModuleView.xhtml), accessed on 1 October 2023.

### 2.9. Quantitative Real-Time PCR (qRT-PCR) Assays

The RNA of HUVECs was extracted by FreeZol Reagent (Vazyme, Cat. No. R711-01, Nanjing, China). After the RNA concentration was detected, RNA was reversed to cDNA by Gradient PCR instrument (Eppendorf, Germany), and further qRT-PCR assays were performed by QuantStudio^TM^ 6 Flex Real-Time PCR System (ABI, Los Angeles, CA, USA). The primer sequences used were as follows: β-actin, forward: CTCGCCTTTGCCGATCC and reverse: TTCTCCATGTCGTCCCAGTT. ICAM-1, forward: AGTGACCATCTACAGCTTTCCG and reverse: CCCCATTCAGCGTCACCTT. GPX4, forward: GAGATCAAAGAGTTCGCCGC and reverse: TGTCGATGAGGAACTTGGTGA. SLC7A11, forward: AAGGTGCCACTGTTCATCCC and reverse: ATGTTCTGGTTATTTTCTCCGACA. SLC3A2, forward: GGGGACGACTCAGGCACC and reverse: CCAGACCATTCTTCTCGGCT. ACSL4, forward: ATACCTGGACTGGGACCGAA and reverse: GCTGGACTGGTCAGAGAGTG. ALOX15, forward: GGGGCAAGGAGACAGAACTC and reverse: GCGGTAACAAGGGAACCTGA. LPCAT3, forward: TGAGCCTTAACAAGTTGGCGA and reverse: GTGGTAGAGCTGGTTTCCAAAGT. CD36, forward: ACGCTGAGGACAACACAGTC and reverse: TGCCACAGCCAGATTGAGAA. Gene expressions were calculated using the 2^−ΔΔCt^ method.

### 2.10. Western Blot Analysis

The protein of HUVECs was extracted using RIPA lysis buffer (Beyotime Biotechnology, Shanghai, China) and the concentration of proteins was detected by BCA assay (Thermo Fisher Scientific, Waltham, MA, USA). Proteins were separated using Sodium dodecyl sulfate-polyacrylamide gel electrophoresis (SDS-PAGE, Epizyme, Shanghai, China) and then transferred to 0.22 μm PVDF membranes (Merck, Darmstadt, Germany). Next, they were blocked with 5% milk in 1 × TBST (Epizyme, Shanghai, China). The primary antibodies against ZO-1 (1:1000, A11417, ABclonal, Woburn, MA, USA), VE-cadherin (1:500, A0734, ABclonal), Occludin (1:1000, A2601, ABclonal), p-eNOS (1:1000, AP0515, ABclonal), ICAM-1 (1:1000, ET1609-46, HUABIO), p-cPLA2 (1:1000, AP0968, ABclonal), cPLA2 (1:1000, A0394, ABclonal), ACSL4 (1:1000, A6826, ABclonal), ALOX15 (1:1000, A6864, ABclonal), LPCAT3 (1:1000, A17604, ABclonal), GPX4 (1:10,000, ET1706-45, HUABIO), SLC7A11 (1:2000, A13685, ABclonal), β-actin (1:20,000, 66009-1-Ig, Proteintech, San Diego, CA, USA) were used to incubate with membranes overnight at 4℃. After washing the membrane with TBST six times and the secondary antibody (HRP Goat anti-Mouse/Rabbit IgG, 1:5000, SA00001-2/SA00001-1, Proteintech) for 1 h at room temperature. Eventually, the band of combined antibodies was visualized by the ECL chemiluminescence instrument (Tanon 5200, Tanon, Shanghai, China) with the chemiluminescence detection kit (Millipore, Burlington, MA, USA). The gray value was analyzed by Image-J 1.52v software.

### 2.11. Ferrous Iron Content in Cells

The HUVECs were incubated with 1 μmol/L FerroOrange (DOJINDO chemical technology Co., Ltd., Shanghai, China) working solution in 37 °C, 5% CO_2_ incubator for 30 min, and then observed under a fluorescence microscopy (Zeiss AX10, Oberkochen, Germany).

### 2.12. Detection of MDA and GSH

The contents of malondialdehyde (MDA) glutathione (GSH) were, respectively, determined with ‘Lipid Peroxidation MDA Assay Kit’ and ‘Total Glutathione Peroxidase Assay Kit’ (S0131S/S0052, Beyotime Biotechnology, Shanghai, China) according to the manufacturer’s instructions.

### 2.13. Immunofluorescence Staining of CD36

After Ang Ⅱ treatment, the cells were washed with PBS buffer solution, and 4% paraformaldehyde was used to fix the cells. The following steps were consistent with the previous study [24]. The nucleus stained by DAPI was blue under excitation, and the positive expression of CD36 was green.

### 2.14. Construction of CD36 Over-Expression Cell Line

The CD36 plasmid vector (PGMLV-CMV-H_CD36-3 × Flag-T2A-Puro) and control plasmid vector (PGMLV-PGK-Puro) were manufactured by and purchased from Genomeditech (Shanghai, China). The CD36 plasmid vector is shown in Figure S1. According to the instructions of the lentivirus manufacturer, the CD36 plasmid and empty vector were, respectively, transfected into HUVEC cells. About 5 × 10^4^ cells were seeded into each well of a 24-well plate. The volume of lentivirus (μL) required per well was calculated based on an infection concentration of MOI (Multiplicity of infection) = 40 and the original lentivirus titer. The lentivirus and DMEM mixture (total 250 μL) were added into each well, and it was cultured in the incubator (37 °C, 5% CO_2_) for 16–24 h. After changing the culture medium and continuing to culture for 48 h, the uninfected cells were killed with 2 μg/mL puromycin, and part of those cells were collected to determine the expression of the target gene and protein to verify the efficiency of over-expression, while the rest cells were used for further experiments or cryopreservation.

### 2.15. Statistical Analysis

All the data were listed as Mean ± SD and visualized by GraphPad Prism 8. The ANOVA method was used to compare multiple groups of data, and LSD analysis was used to compare the two groups via SPSS 26.0 software. Data are representative of at least three independent experiments and biological replicates. Differences between groups were statistically significant at *p*-value < 0.05.

## 3. Result

### 3.1. Ang II Caused the Vascular Endothelial Dysfunction

**Figure 1:**
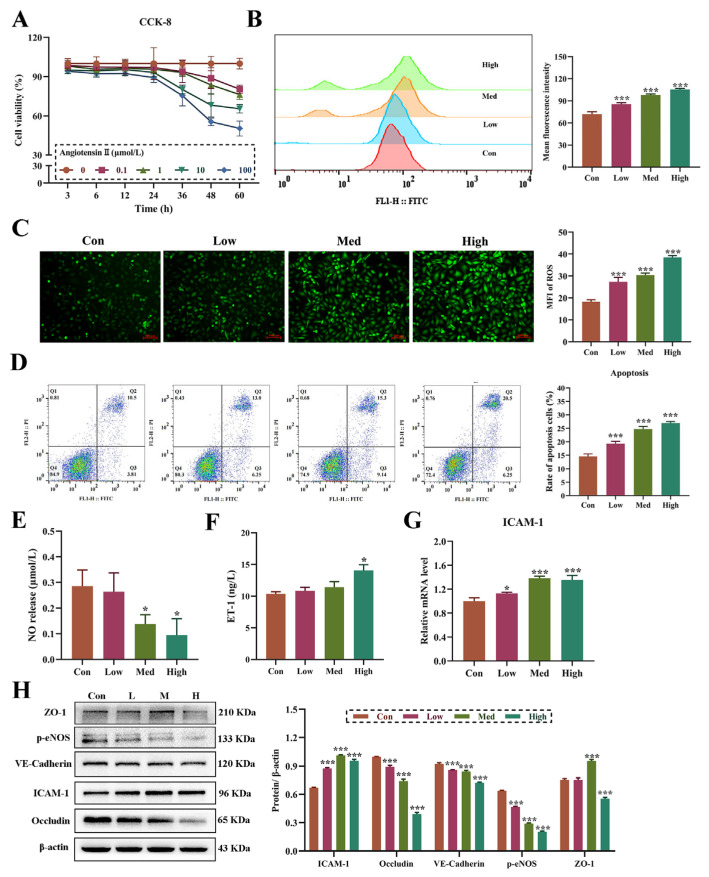
Ang Ⅱ induces endothelial dysfunction in HUVECs. (**A**): CCK-8 analysis of cells treated with 0.1, 1, 10, 100 μM Ang Ⅱ for 3, 6, 12, 24, 36, 48, 60 h. (**B**): Flow cytometry detection of cellular ROS production. (**C**): Fluorescence microscope detection of cellular ROS production. Magnification 100×, Scale bar 100 μm. (**D**): Flow cytometry analysis of cell apoptosis rate (%). (**E**): The detection of NO concentration released from cells. (**F**): The synthesis of Endothelin-1 (ET-1) detected by ELISA assays. (**G**): The mRNA expression of ICAM-1. (**H**): The protein expression of ZO-1, p-eNOS, VE-cadherin, Occludin and ICAM-1. The original WB images are shown in Figure S2. One-way ANOVA was used to evaluate the statistical significance of differences, n ≥ 3 replicates. * *p* < 0.05, *** *p* < 0.001, compared with the control group.

The cytotoxicity of Ang II on HUVECs was explored from the aspects of time effect and dose effect. The results showed that the cell viability decreased significantly with the increase in concentration and exposure time of Ang II. According to the results of CCK-8, there was no significant difference in the effect of 0.1 and 1 μmol/L Ang II on HUVECs viability. Moreover, after 48 h of treatment, compared with the control group, the cell viability of each group decreased significantly (Figure 1A). Therefore, 0.1, 10, and 100 μmol/L were determined to be the low, medium, and high concentrations, and 48 h was the exposure time. The results of flow cytometry showed that exposure to 0.1–100 μmol/L Ang II for 48 h significantly increased ROS production (Figure 1B,C) and the rate of cell apoptosis (Figure 1D). At the same time, Ang II significantly reduced the secretion of nitric oxide (Figure 1E) and the expression of p-eNOS (Figure 1H) and increased the synthesis of ET-1 (Figure 1F), indicating that Ang II caused the imbalance of vasodilation and vasoconstriction homeostasis of vascular endothelial. The effect of Ang II on the barrier and adhesion function-related molecules of HUVECs was further explored, and the results showed that the adhesion factor ICAM-1 was significantly up-regulated (Figure 1G,H). Western blot results showed that the expression of endothelial barrier-related proteins, VE-cadherin and Occludin were down-regulated in a dose-dependent manner, and the ZO-1 protein was significantly down-regulated at high concentration (Figure 1H).

### 3.2. Ang II Impaired Fatty Acid Metabolic Homeostasis in Vascular Endothelial Cells

**Figure 2:**
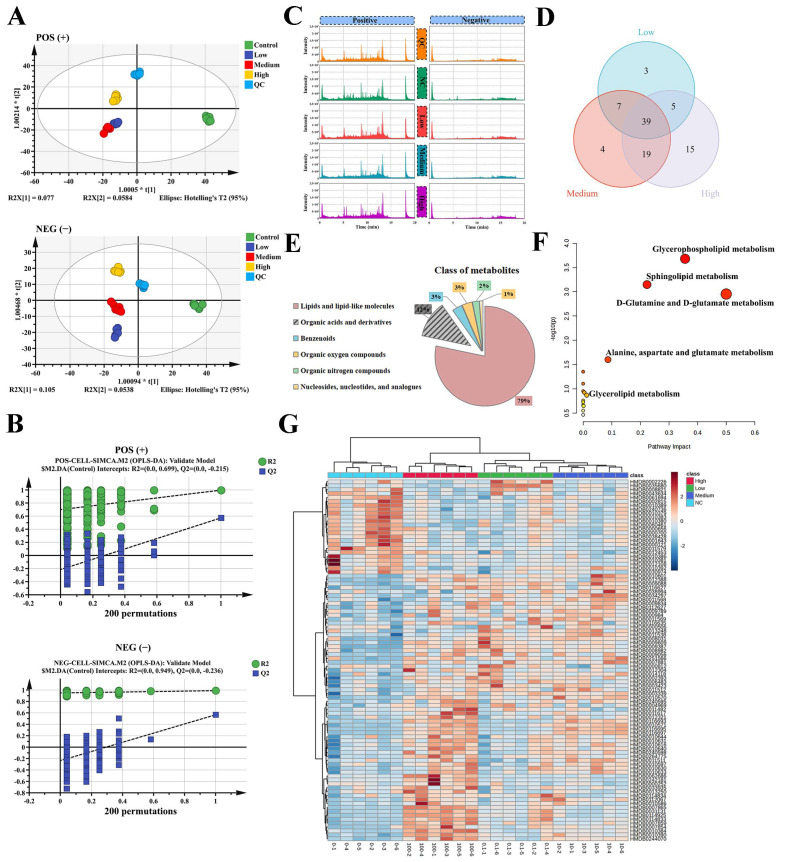
Ang Ⅱ induces lipid metabolism disorder in HUVECs. (**A**): The OPLS-DA analysis of positive ion (R^2^Y = 0.753, Q^2^ = 0.440) and the OPLS-DA analysis of negative ion (R^2^Y = 0.982, Q^2^ = 0.401). (**B**): The 200 permutations test model of positive (R^2^ = 0.699, Q^2^ = −0.215) and negative (R^2^ = 0.949, Q^2^ = −0.236) ion. (**C**): Metabolic peak spectra of positive and negative ions. (**D**): Venn diagram of SDMs in low, medium and high-concentration groups. (**E**): The classification of SDMs. (**F**): The KEGG analysis of differential metabolic pathway. (**G**): Heat map of differential metabolites. One-way ANOVA or Kruskal–Wallis H were used to evaluate the statistical significance of differences, n = 6 replicates.

In order to further explore the molecular mechanism of Ang II damaging vascular endothelial function, Liquid Chromatograph Mass Spectrometer (LC-MS) technology was used to analyze the non-target metabolomics of HUVECs induced by low, medium and high concentrations of Ang II. The score plot of the PCA model in positive and negative ion modes showed that there was a significant difference between the Ang II exposure group and the control group (Figure S3A,B). The score plot of the OPLS-DA model showed obvious separation between cells in different concentration groups, showing good model fitting and prediction performance (Figure 2A). In addition, the validation of the model was evaluated by 200 permutation tests, and the results showed that the model had good predictability (Q^2^ < 0, Figure 2B). The metabolic peaks of each group are shown in Figure 2C. Among them, 54 differential metabolites were found in the low group, 69 differential metabolites in the medium group, and 78 differential metabolites in the high group, showing a dose-dependent manner (Figure 2D). In the classification analysis of all SDMs, we found that lipids or lipid-like molecules accounted for 79% of all differential metabolites (Figure 2E). The results of KEGG pathway enrichment showed that differential metabolites were mainly enriched in the Glycerophospholipid metabolism, Sphingolipid metabolism, Glutamine-glutamic acid metabolism, Alanine-aspartate-glutamic acid metabolism, Phospholipid metabolism and Linoleic acid metabolism pathways (Figure 2F). The differential expression heat map of SDMs is shown in Figure 2G. We further obtained the relative peak density values of phospholipid PUFAs (PL–PUFAs) from the SDMs. The results are shown in Figure S2C. Compared with the control group, the relative peak densities of PE (22:4), PE (20:4/18:0), PC (18:0/14:0) and PC (14:0/20:3) in the Ang II exposure group were significantly increased.

### 3.3. Ang II Induced Lipid Peroxidation-Mediated Ferroptosis in HUVECs

**Figure 3:**
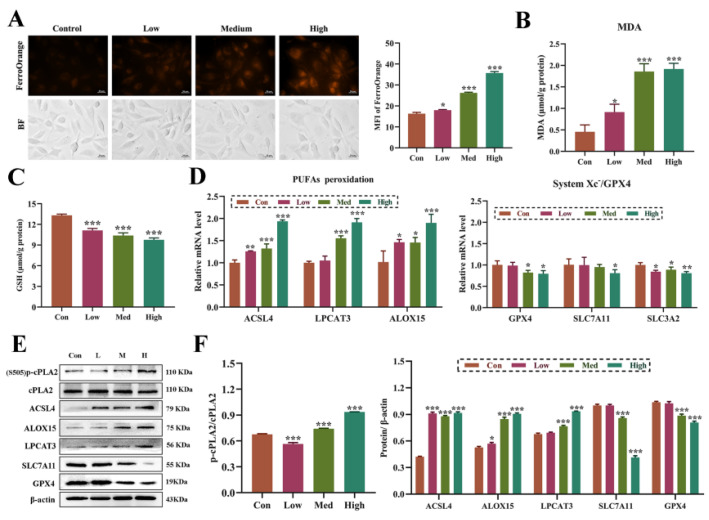
Ang Ⅱ activates ferroptosis in HUVECs. (**A**): The binding of FerroOrange probe to intracellular Fe^2+^ observed under a microscope. Magnification 400×, scale 20 μm. (**B**): Intracellular malondialdehyde (MDA) content. (**C**): Total intracellular glutathione (GSH) content. (**D**): The expression of mRNA involved in PUFAs peroxidation and System Xc-/GPX4. (**E**): The expression of a protein involved in PUFAs peroxidation and System Xc-/GPX4. The original WB images are shown in Figure S4. (**F**): The gray value analysis of protein bands via Image-J 1.52v software. One-way ANOVA was used to evaluate the statistical significance of differences, n = 3 replicates. * *p* < 0.05, ** *p* < 0.01, *** *p* < 0.001, compared with control group.

In order to investigate whether the abnormal metabolism of phospholipid polyunsaturated fatty acids in HUVECs induced by Ang II is mediated by iron-dependent lipid peroxidation, the intracellular free ferrous ion content was detected. Figure 3A showed that Ang II significantly increased the level of Fe^2+^ in the cells compared with the control group, and the level of lipid peroxidation showed a dose-dependent increase (Figure 3B), and glutathione levels showed a downward trend (Figure 3C). The key signaling pathways related to ferroptosis were further detected. The results showed that Ang II activated polyunsaturated fatty acid peroxidation signals, significantly upregulated the mRNA (Figure 3D) and protein (Figure 3E,F) expression of ACSL4, LPCAT3 and ALOX15, and induced an increase in the phosphorylation level of cPLA2 (Figure 3E,F). In addition, Ang II inhibited the antioxidant system and significantly down-regulated the expression of GPX4, SLC7A11 and SLC3A2 (Figure 3D–F).

### 3.4. Fer-1 Reversed Ang II-Induced Ferroptosis in HUVECs

**Figure 4:**
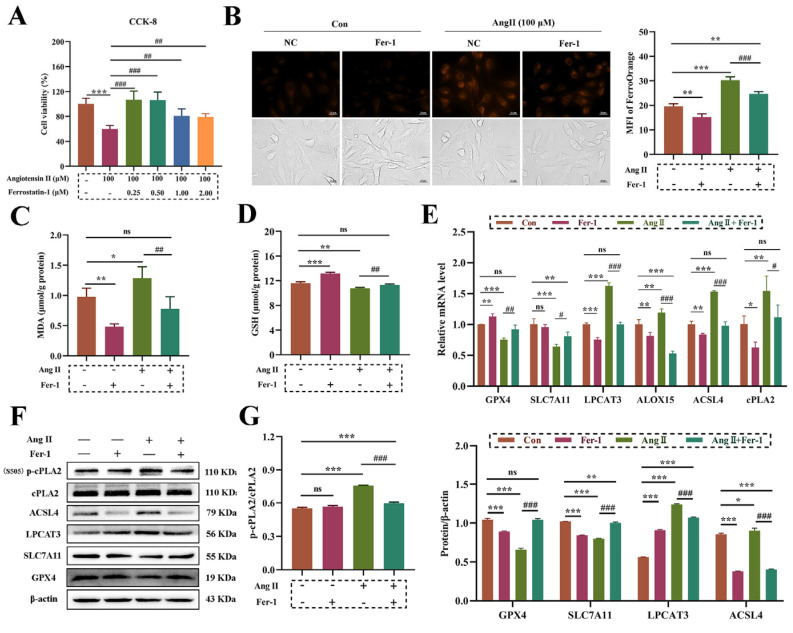
Fer-1 rescues ferroptosis induced by Ang Ⅱ in HUVECs. (**A**): CCK-8 analysis of cells treated with 100 μM Ang Ⅱ (with or without 1 μM Fer-1) for 48 h. (**B**): The binding of FerroOrange probe to intracellular Fe^2+^ observed under a microscope. Magnification 400×, scale 20 μm. (**C**): Intracellular malondialdehyde (MDA) content. (**D**): Total intracellular glutathione (GSH) content. (**E**): The expression of mRNA involved in PUFAs peroxidation and System Xc-/GPX4. (**F**): The expression of a protein involved in PUFAs peroxidation and System Xc-/GPX4. The original WB images are shown in Figure S5. (**G**): The gray value analysis of protein bands via Image-J 1.52v software. One-way ANOVA was used to evaluate the statistical significance of differences, and LSD analysis was used to compare between two groups. n = 3 replicates. * *p* < 0.05, ** *p* < 0.01, *** *p* < 0.001, compared with control group, ^#^ *p* < 0.05, ^##^ *p* < 0.01, ^###^ *p* < 0.001, compared with 100 μM Ang Ⅱ group.

Fer-1 is a highly effective ferroptosis inhibitor. We determined whether Fer-1 inhibits Ang II-induced ferroptosis in HUVECs. The CCK-8 results showed that 250 and 500 nM Fer-1 treatment significantly increased cell proliferation activity, and 1 and 2 μM fer-1 partially reversed the decrease in cell proliferation activity induced by 100 μM Ang II (Figure 4A). It has been reported that Fer-1 can play a role in inhibiting ferroptosis by reducing lipid peroxidation and the content of unstable iron [25]; therefore, related indicators were further examined. The results of 1 μM Fer-1 treatment with 100 μM showed that Fer-1 significantly reduced Fe^2+^ overload (Figure 4B) and Ang II-induced lipid peroxidation (Figure 4C) in HUVECs. Similarly, Fer-1 reversed Ang II-induced GSH degradation (Figure 4D). The results of qRT-PCR and Western Blot showed that Fer-1 reversed the down-regulation of the System Xc-/GPX4 signaling and the activation of the PUFA peroxidation signaling pathway (Figure 4E–G).

### 3.5. Inhibition of Ferroptosis Rescued Ang II-Induced Vascular Endothelial Dysfunction

**Figure 5:**
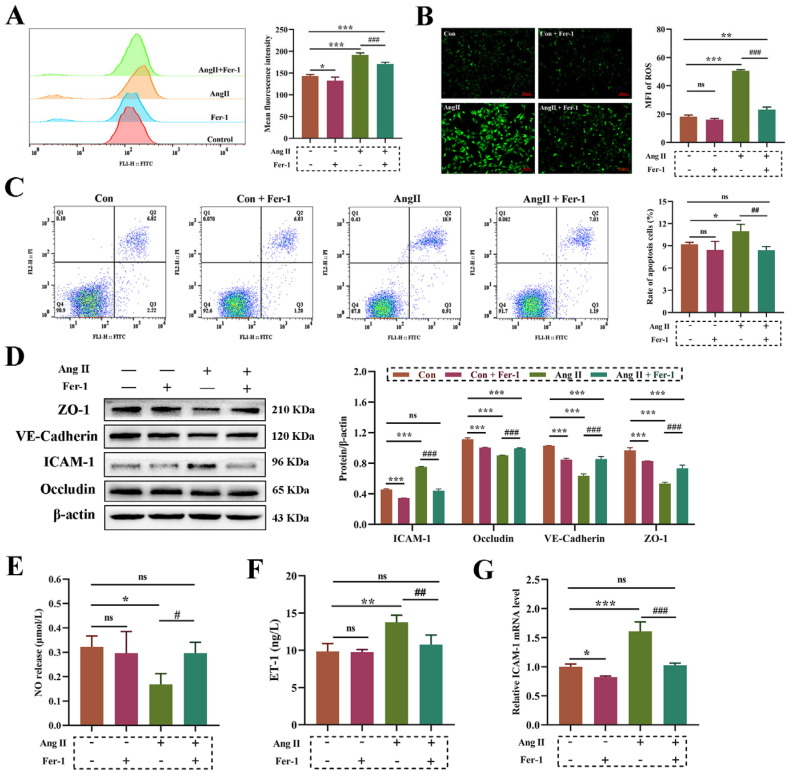
Ang Ⅱ induces vascular endothelial dysfunction by promoting ferroptosis in HUVECs. (**A**): Flow cytometry detection of cellular ROS production. (**B**): Fluorescence microscope detection of cellular ROS production. Magnification 100×, Scale bar 100 μm. (**C**): Flow cytometry analysis of cell apoptosis rate (%). (**D**): The protein expression of ZO-1, VE-cadherin, Occludin and ICAM-1. The original WB images are shown in Figure S6. (**E**): The detection of NO concentration released from cells. (**F**): The synthesis of Endothelin-1 (ET-1) detected by ELISA assays. (**G**): The mRNA expression of ICAM-1. One-way ANOVA was used to evaluate the statistical significance of differences, and LSD analysis was used to compare between two groups. n = 3 replicates. * *p* < 0.05, ** *p* < 0.01, *** *p* < 0.001, compared with control group, ^#^ *p* < 0.05, ^##^ *p* < 0.01, ^###^ *p* < 0.001, compared with 100 μM Ang Ⅱ group.

Fer-1 was used to further explore whether lipid peroxidation-mediated ferroptosis is involved in Ang II-induced vascular endothelial dysfunction. Figure 5A,B shows that the increase in ROS production induced by Ang II was significantly reversed by Fer-1 (1 μM). The results of flow cytometry analysis showed that there was no significant difference in the level of apoptosis rate between the Fer-1 and Ang II co-treated group and the control group, which proved that Fer-1 reversed the increase in Ang II-induced apoptosis rate (Figure 5C). In addition, the degradation of key molecules of endothelial cell barrier function (ZO-1, VE-cadherin and Occludin) induced by Ang II was also alleviated by Fer-1 treatment (Figure 5D). The decrease in NO (Figure 5E), the increase in ET-1 (Figure 5F) and the up-regulated expression of ICAM-1 (Figure 5G) in the Ang II group were also restored to the level of the control group after Fer-1 intervention.

### 3.6. Ang II Activated CD36 in HUVECs

**Figure 6:**
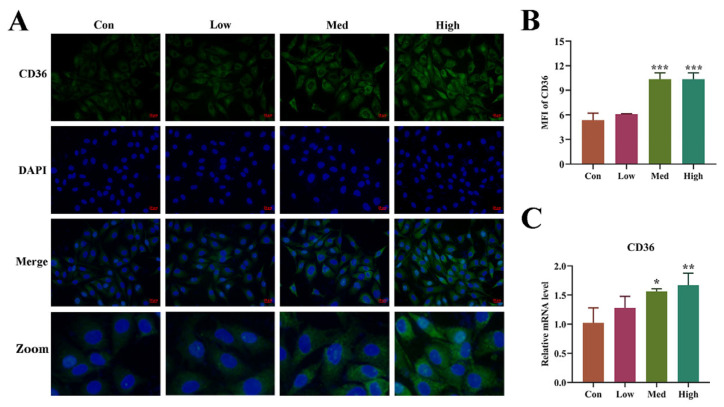
Ang Ⅱ upregulates CD36 expression in HUVECs. (**A**): The representative images of immunofluorescence (IF) assay. Blue, DAPI, Nucleus. Green, FITC, CD36 positive cells. Magnification 400×, scale 20 μm. (**B**): The mean fluorescence intensity of CD36(+) cells. (**C**): The expression of mRNA detected by qRT-PCR. One-way ANOVA was used to evaluate the statistical significance of differences, n = 3 replicates. * *p* < 0.05, ** *p* < 0.01, *** *p* < 0.001, compared with control group.

After Ang II treatment for 48 h, the expression level of CD36 in the cells was further detected. The results of immunofluorescence (IF) showed that CD36 showed a dose-dependent up-regulated, and the activated CD36 was mainly located in the cytoplasm (Figure 6A,B). At the same time, the level of CD36 mRNA was consistent with the results of the IF assay (Figure 6C). These results indicated that Ang Ⅱ activated the expression of CD36 in HUVECs.

### 3.7. CD36 Participates Ang II-Induced Ferroptosis and Vascular Endothelial Dysfunction

**Figure 7:**
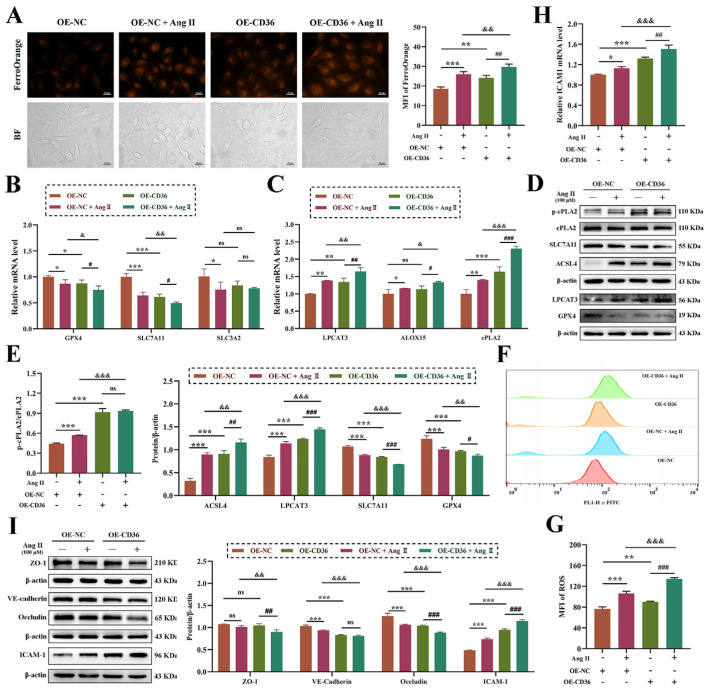
CD36 is involved in Ang II-induced ferroptosis and dysfunction of HUVECs. (**A**): The binding of FerroOrange probe to intracellular Fe^2+^ observed under a microscope. Magnification 400×, scale 20 μm. (**B**): The expression of mRNA involved in System Xc-/GPX4. (**C**): The expression of mRNA involved in PUFAs peroxidation. (**D**): The expression of a protein involved in PUFAs peroxidation and System Xc-/GPX4. The original WB images are shown in Figure S8. (**E**): The gray value analysis of protein bands via Image-J 1.52v software. (**F**): Flow cytometry detection of cellular ROS production. (**G**): The mean fluorescence intensity of ROS. (**H**): The expression of ICAM-1 mRNA detected by qRT-PCR. (**I**): The protein expression of ZO-1, VE-cadherin, Occludin and ICAM-1. The original WB images are shown in Figure S9. One-way ANOVA was used to evaluate the statistical significance of differences, and LSD analysis was used to compare between two groups. n = 3 replicates. * *p* < 0.05, ** *p* < 0.01, *** *p* < 0.001, compared with OE-NC group, ^#^ *p* < 0.05, ^##^ *p* < 0.01, ^###^ *p* < 0.001, compared with OE-CD36 group, ^&^ *p* < 0.05, ^&&^ *p* < 0.01, ^&&&^ *p* < 0.001, compared with the group of OE-NC cells treated with 100 μM Ang Ⅱ.

In order to further explore whether CD36 is involved in the regulation of Ang II-induced ferroptosis and vascular endothelial dysfunction, cell lines of OE-CD36 and OE-NC were constructed. The results of mRNA and protein verification are shown in Figure S7, we used OE-NC and OE-CD36 cell lines for in-depth exploration. Firstly, compared with the OE-NC group, the content of Fe^2+^ in the OE-CD36 group was significantly increased, and, compared with the Ang II-treated OE-NC cells, the content of Fe^2+^ in the OE-CD36 cells treated with Ang II was higher (Figure 7A). In addition, Ang II-induced degradation of GPX4 and SLC7A11, phosphorylation of cPLA2, and the increased expression of ACSL4, LPCAT3 and ALOX15 were further aggravated by over-expression of CD36 (Figure 7B–E). These results suggested that CD36 may be involved in the regulation of Ang II-induced lipid peroxidation-mediated ferroptosis in HUVECs. Whether CD36 has a regulatory effect on endothelial dysfunction has been confirmed in the following results. Ang II-mediated ROS production was significantly increased in CD36 over-expressing cells (Figure 7F,G). Ang II-induced endothelial barrier dysfunction and up-regulation of adhesion factor ICAM-1 expression were more significant in the OE-CD36 group (Figure 7H,I). These results suggested that CD36 may be involved in the regulation of Ang II-induced endothelial dysfunction in HUVECs.

## 4. Discussion

Hypertension is the leading cause of premature death and CVD worldwide [26,27]. Endothelial cells are an important part of blood vessels and are crucial for vascular health. The functions of vasodilation, vasoconstriction, permeability and monocyte adhesion are regulated by endothelial cells [28]. Endothelial dysfunction is not only an important mechanism in the development of hypertension but also an early signal of various cardiovascular diseases [29]. A recent study has revealed that early endothelial function improvement can help prevent subclinical target organ damage (STOD) in patients with hypertension [30]. Therefore, this study aims to explore the potential molecular targets and signaling pathways of hypertension-induced vascular endothelial dysfunction and provide a molecular basis for the development of preventive drugs for hypertension-induced cardiovascular diseases. Our study constructed a hypertensive ECs injury model by Ang II. Hypertension causes HUVEC cytotoxicity by increasing ROS content and apoptosis rate. Vasodilation and vasoconstriction function-related molecules are affected by Ang II along with down-regulation of p-eNOS, reducing NO synthesis and increasing ET-1 synthesis. At the same time, the adhesion ability-related protein ICAM-1 is up-regulated. These results are consistent with the results of other studies [2,6,31,32,33]. In addition, Ang II damages the endothelial barrier-related proteins ZO-1, VE-cadherin and Occludin.

Metabonomics is a rapidly developing field in system biology. It mainly studies cells and biological serum and measures the metabolic changes of biological samples in the process of disease development [34,35]. LC-MS provides the most compatible technology for the sensitive detection of small molecule metabolites with strong reliability and reproducibility [36,37]. In 2008, Akira et al. analyzed the urine samples of six 8-week-old spontaneously hypertensive rats (SHR) by NMR metabolomics. The results showed that citrate and α-ketoglutarate in the urine of SHR rats were significantly lower than those of normotensive rats, suggesting that the early metabolic process of hypertension has begun to change [38]. At the same time, Lu et al. found, in the plasma of SHR rats at 10–18 weeks, that, with the increase in blood pressure in rats, the levels of free fatty acids (FFAs) such as oleic acid, linoleic acid and stearic acid in the plasma gradually increased [39]. This indicates that metabolic abnormalities are involved in the occurrence and development of hypertension. In order to further explore the possible molecular mechanism of hypertension-induced vascular endothelial dysfunction, we used LC-MS technology to reveal the metabolic profile changes of HUVECs induced by Ang Ⅱ for the first time. The glycerophospholipid metabolism, sphingolipid metabolism, glutamine–glutamate metabolism, alanine–aspartate–glutamate metabolism, phospholipid metabolism and linoleic acid metabolism pathways were the main changed metabolic pathways. In particular, ferroptosis is reported to be driven by iron-dependent phospholipid peroxidation [10,40]. PUFAs are the substrates of phospholipid peroxidation. Therefore, the metabolic disorder of glycerophospholipids is closely related to the mechanism of ferroptosis. It has been reported that PEs with arachidonic tails (PE-AA, 20:4) or adrenic tails (PE-AdA, 22:4) are the main PL–PUFAs that are oxidized during ferroptosis [41]. Our metabolomics results also found a significant increase in metabolic intensity of PE (22:4) and PE (20:4/18:0). Therefore, we hypothesized that Ang II may be involved in mediating vascular endothelial dysfunction through lipid peroxidation-mediated ferroptosis.

Based on the above assumptions, we explored the ferroptosis signaling pathway. Liu et al. first discovered that Ang II may regulate ferroptosis in HUVECs through the p53-ALOX12 signaling axis [42]. The existing in vitro evidence of Ang II and ferroptosis is very limited, especially the results of Ang II on the abnormal phospholipid metabolism of HUVECs are pioneering. The cystine/glutamate transporter system (Xc- system) is composed of two proteins, light chain SLC7A11 and heavy chain SLC3A2 [43]. As a chaperone protein of SLC7A11, SLC3A2 is used to maintain the stability of the SLC7A11 protein. SLC7A11 mediates the exchange of cystine and glutamate on the cell membrane, promotes the synthesis of GSH and protects cells from oxidative stress damage [40]. GPX4 is known to be a key monitoring mechanism of ferroptosis, which inhibits the occurrence of ferroptosis mainly by catalyzing the antioxidant effect of GSH [9]. Our data showed that Ang II inhibited the expression of SLC7A11/SLC3A2 and GSH synthesis and further down-regulated the expression of GPX4. Ferroptosis inhibitor Fer-1 significantly reversed the degradation of the GPX4/Xc- system induced by Ang II. The accumulation of lipid peroxides induced by Ang II may depend on another widely recognized signaling pathway, the ACSL4/LPCAT3/ALOX15 signaling cascade, which was significantly activated in our research. The ACSL4 and LPCAT3 are key drivers of ferroptosis, and these enzymes can activate endogenous iron chains through lipid metabolism reprogramming [44]. ACSL4 catalyzes the entry of PUFAs into phospholipids to synthesize PL–PUFAs, which are then directly oxidized by ALOX15, thereby promoting ferroptosis and further affecting cellular lipid synthesis [45]. We found that Ang II-induced ACSL4/LPCAT3/ALOX15 activation and increased MDA synthesis were partially reversed by Fer-1.

In addition, we further explored the relationship between ferroptosis and hypertension-induced vascular endothelial dysfunction. The results showed that Fer-1 partially rescued Ang II-induced HUVECs apoptosis, increased ROS synthesis, increased adhesion factor ICAM-1, impaired endothelial barrier and vasodilation/vasoconstriction-related molecules. In vivo studies have shown that GPX4 over-expression inhibits the development of atherosclerosis by reducing the expression of endothelial cell adhesion molecules and up-regulation of endothelial necrosis and apoptosis [46]. Inhibition of ACSL4 can improve atherosclerosis by inhibiting vascular endothelial ferroptosis [47]. This evidence reveals the potential value of ferroptosis inhibitors in the prevention of cardiovascular disease.

CD36 is a scavenger receptor in a variety of immune cells and non-immune cells, which can regulate signal transduction and transport of long-chain FFAs [48]. At present, there is no evidence that Ang II directly induces CD36 activation. Our results showed that Ang II induced an increase in CD36 expression in a dose-dependent manner. Studies have shown that CD36-mediated fatty acid uptake and transport leads to lipid peroxidation and ferroptosis [19]. Accordingly, we suspected that CD36 is probably involved in the Ang II-induced ferroptosis of HUVECs by inducing lipid peroxidation. Therefore, we constructed a new cell line with CD36 over-expression in HUVECs. Our results were consistent with the hypothesis that Ang II treatment significantly increased the sensitivity of cell lipid peroxidation and ferroptosis after over-expression of CD36 compared with the control cells. A large number of studies have revealed the importance of targeting vascular or endothelial cell CD36 in the treatment of vascular injury and cardiovascular disease [16,49,50]. Our findings further enrich the mechanism of hypertension-induced vascular endothelial dysfunction and possible intervention targets. However, the current evidence is limited to in vitro studies, and in vivo studies are needed to demonstrate the role of EC-CD36-mediated ferroptosis in hypertension-related vascular complications in the future.

In summary, our study preliminarily showed that hypertension induces intracellular PUFAs to bind to phospholipids under the catalysis of ACSL4 and LPCAT3 to form PL–PUFAs, which are further oxidized by ALOX15, resulting in the accumulation of lipid peroxides. This signal is mediated by CD36. In addition, CD36 is involved in the destruction of the system Xc-/GPX4-mediated antioxidant effect. These results provide a new molecular target for hypertension-mediated endothelial dysfunction.

## 5. Conclusions

Ang II induces the injury of HUVECs, decreases vascular diastole and endothelial barrier-related molecules, and increases vascular constriction and adhesion-related molecules, which may be related to CD36 and its mediated lipid peroxidation and ferroptosis signals, including the degradation of system Xc-/GPX4 and the activation of ACSL4/LPCAT3/ALOX15 signaling pathways.

## Data Availability

The authors did not use generative AI or AI-assisted technologies in the development of this manuscript.

## Supplementary Materials

The following supporting information can be downloaded at www.mdpi.com/article/10.3390/biom14111456/s1, Figure S1: The CD36 plasmid vector; Figure S2: The original WB images of Figure 1H; Figure S3: The supplementary materials of Figure 2; Figure S4: The original WB images of Figure 3E; Figure S5: The original WB images of Figure 4F; Figure S6: The original WB images of Figure 5D; Figure S7: The supplementary materials of Figure 7; Figure S8: The original WB images of Figure 7D; Figure S9: The original WB images of Figure 7I.

## Abbreviations

**ACSL**: Acyl-CoA synthetase long-chain ligase; ALOX: Arachidonate lipoxygenase; **Ang II**: Angiotensin II; **CVDs**: Cardiovascular diseases; **CCK-8**: Cell Counting Kit-8; **DCFH-DA**: Dichloro-dihydro-fluorescein diacetate; **DMEM**: Dulbecco’s Modified Eagle Medium; **ECs**: Endothelial cells; **ED**: Endothelial dysfunction; **ET-1**: Endothelin-1; **FAT**: Fatty acid translocase; **FAs**: Fatty acids; **FBS**: Fetal bovine serum; **FFAs**: Free fatty acids; **GSH**: Glutathione; **GPX4**: Glutathione peroxidase 4; **HMDB**: Human Metabolome Database; **HUVECs**: Human umbilical vein endothelial cells; **IF**: Immunofluorescence; **LC-MS**: Liquid Chromatograph Mass Spectrometer; **LDL**: Low-density lipoprotein; **LPCAT**: Lysophosphatidyl-CoA acyltransferase; **MDA**: Malondialdehyde; **NO**: Nitric oxide; **OD**: Optical density; **OPLS-DA**: Orthogonal projection to latent structure discriminate analysis; **PUFAs**: Polyunsaturated fatty acids; **PCA**: Principal component analysis; **PI**: Propidium Iodide; **ROS**: Reactive oxygen species; **RAAS**: Renin-angiotensin-aldosterone system; **SDMs**: Significantly differential metabolites; **SHR**: Spontaneously hypertensive rats; **VED**: Vascular endothelial dysfunction.
