# Supplementary material for: Angiotensin II Induces Vascular Endothelial Dysfunction by Promoting Lipid Peroxidation-Mediated Ferroptosis via CD36
Source: Biomolecules. 2024 Nov 17;14(11):1456. doi: 10.3390/biom14111456 (PMC11591770; doi:10.3390/biom14111456)
Supplement: Supplementary file 1 [file biomolecules-14-01456-s001.zip › biomolecules-3303782-supplementary.pdf]

Supplementary figures

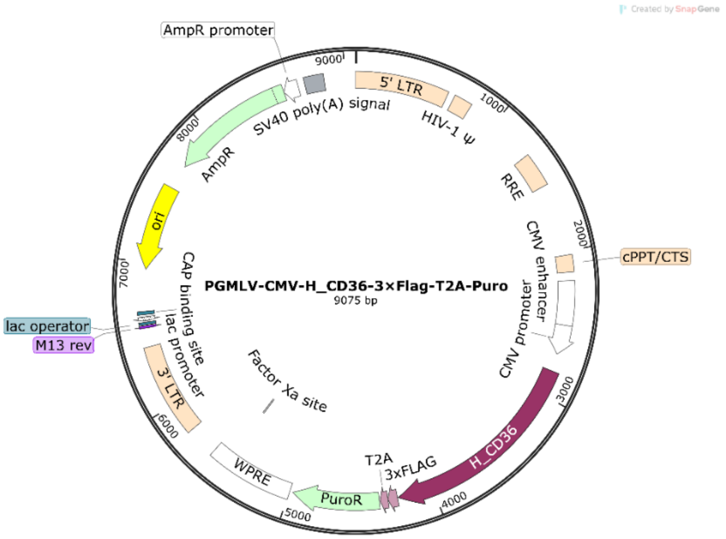

Fig.s1. The CD36 plasmid vector.

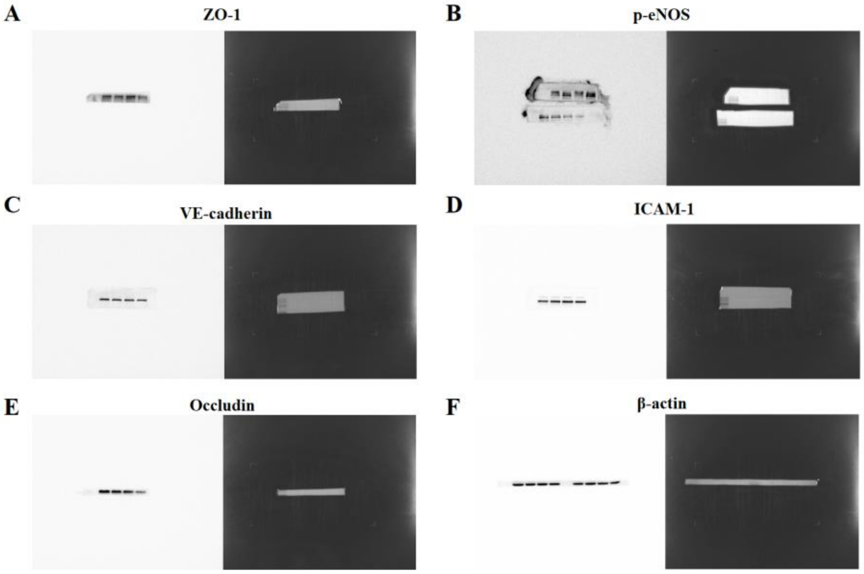

Fig.s2. The original WB images of Fig.1 H.

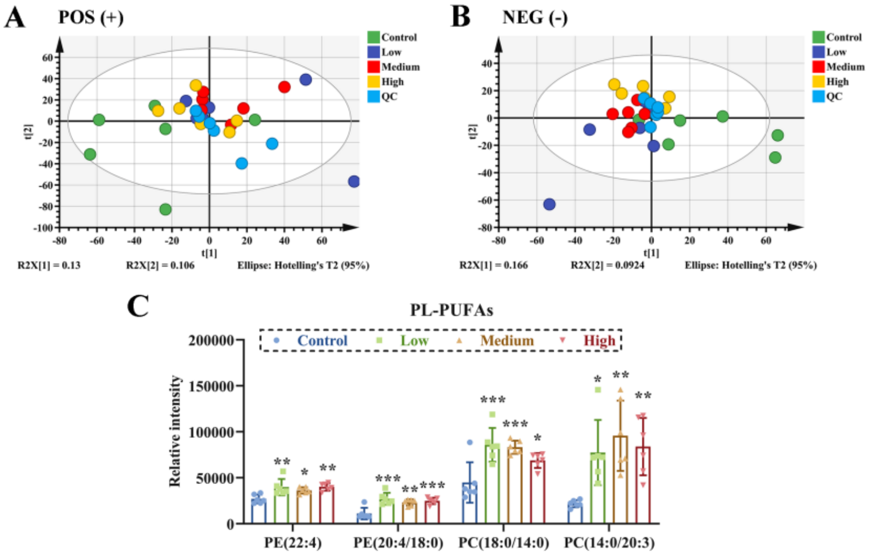

Fig.s3. A: The PCA analysis of positive ion. **B:** The PCA analysis of negative ion. **C:** The relative peak density of PE (22:4), PE (20:4/18:0), PC (18:0/14:0) and PC (14:0/20:3). *$P < 0.05$, **$P < 0.01$, ***$P < 0.001$, compared with control group.

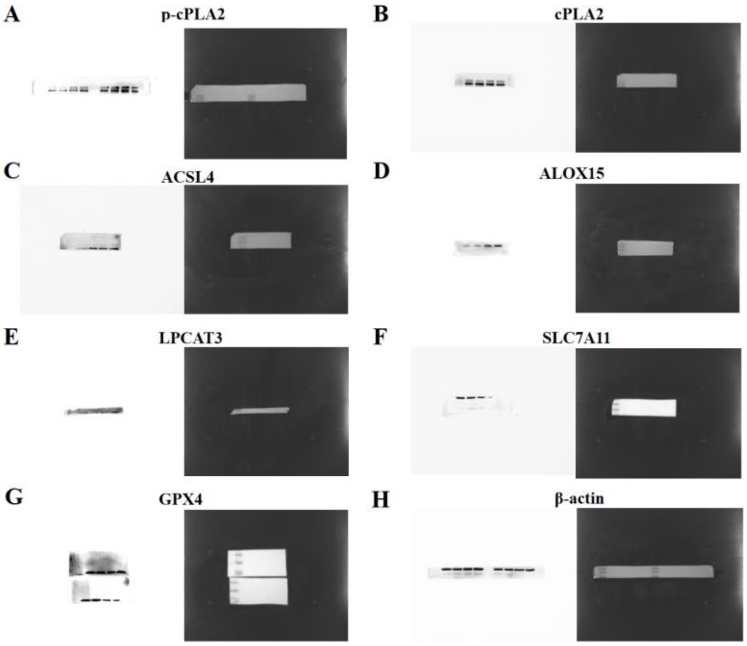

Fig.s4. The original WB images of Fig.3 E.

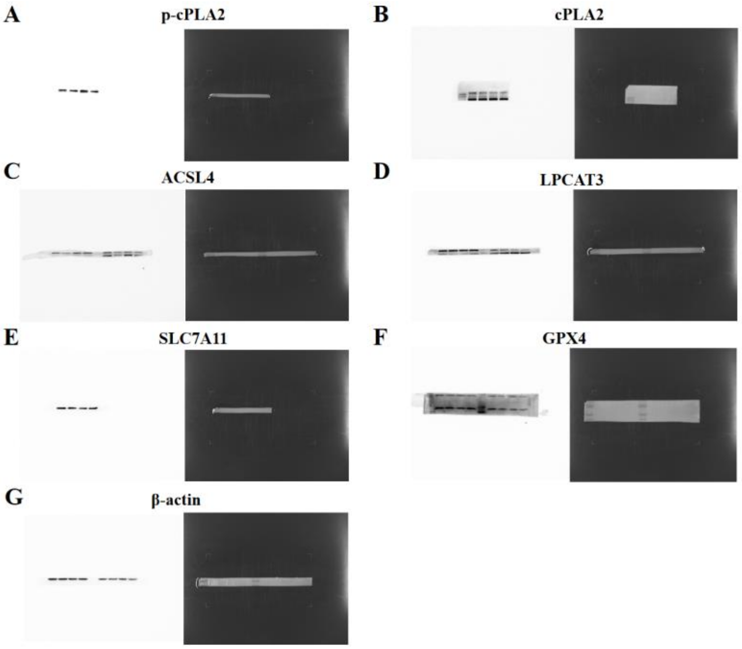

Fig.s5. The original WB images of Fig.4 F.

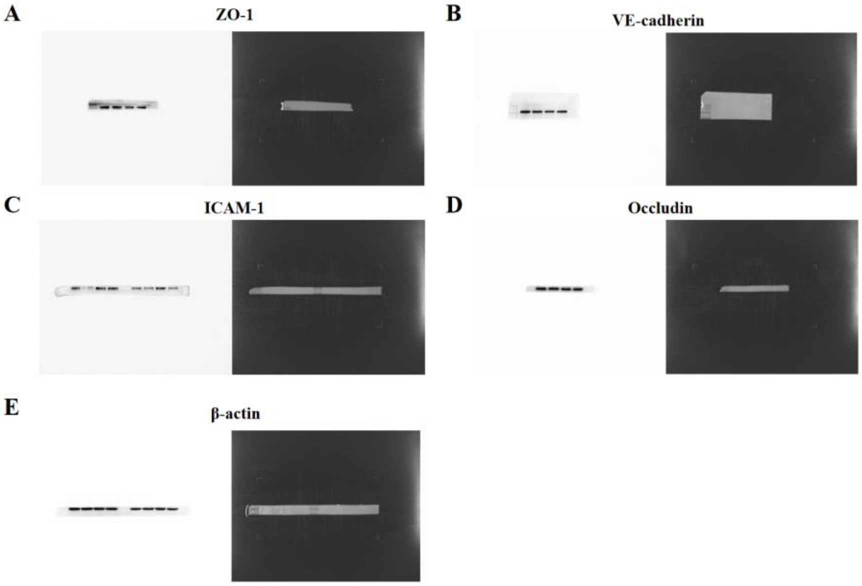

Fig.s6. The original WB images of Fig.5 D.

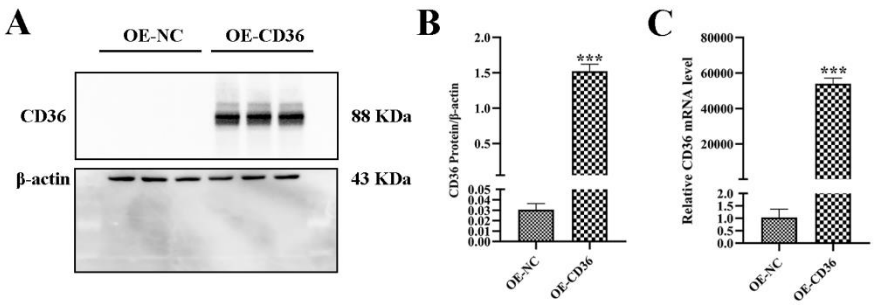

Fig.s7. A: The protein expression of CD36 in over-expressing CD36 group and control group. **B:** The gray value analysis of protein bands via Image-J 1.52v software. **C:** The mRNA expression of CD36 in over-expressing CD36 group and control group. One-way ANOVA was used to evaluate the statistical significance of differences， n = 3 replicates. *$P < 0.05$, **$P < 0.01$, ***$P < 0.001$, compared with OE-NC group.

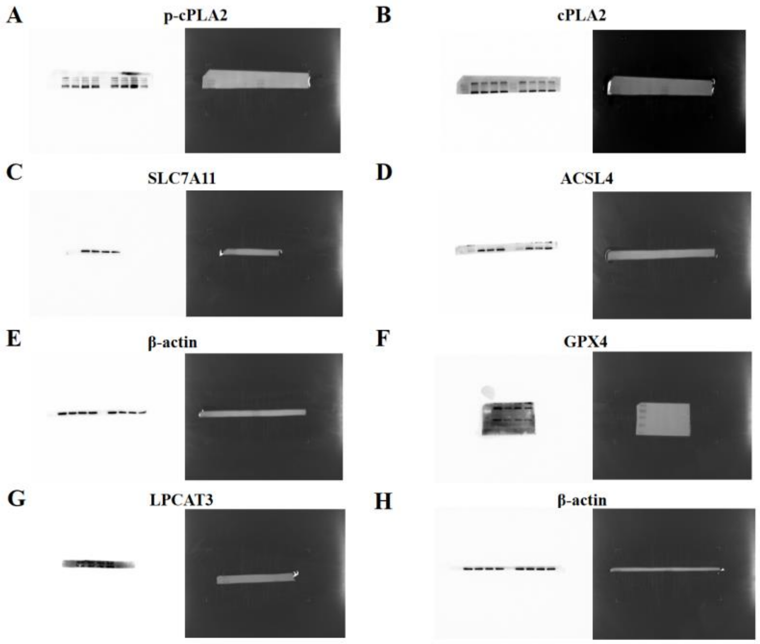

Fig.s8. The original WB images of Fig.7 D.

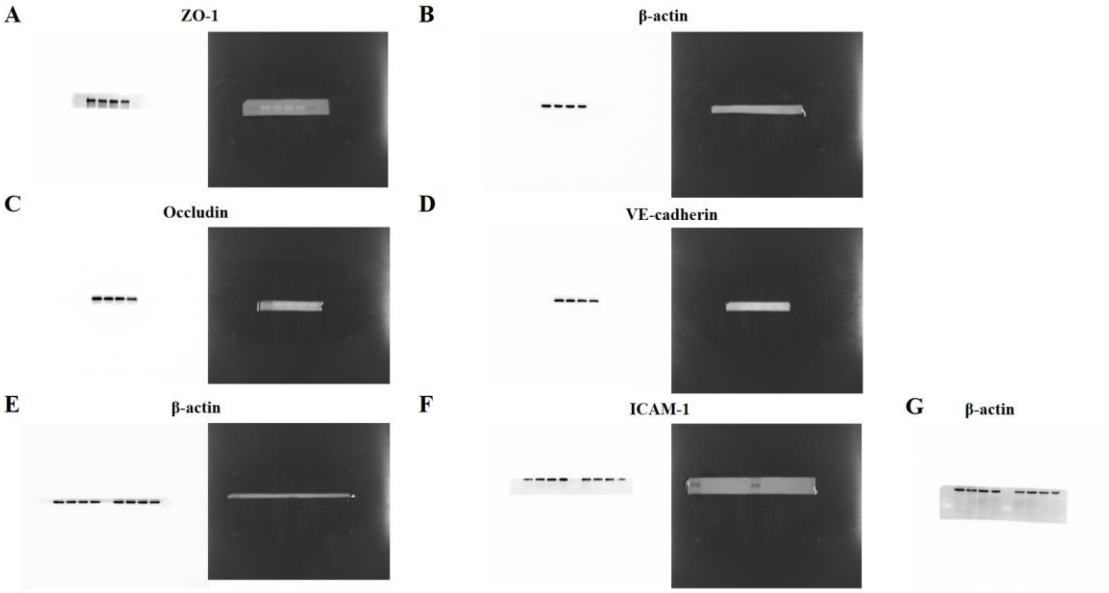

Fig.s9. The original WB images of Fig.7 I.
